# Contributions of Na_V_1.8 and Na_V_1.9 to excitability in human induced pluripotent stem-cell derived somatosensory neurons

**DOI:** 10.1038/s41598-021-03608-x

**Published:** 2021-12-20

**Authors:** Matthew Alsaloum, Julie I. R. Labau, Shujun Liu, Mark Estacion, Peng Zhao, Fadia Dib-Hajj, Stephen G. Waxman

**Affiliations:** 1 Department of Neurology, Yale University School of Medicine, New Haven, CT USA; 2 Center for Neuroscience and Regeneration Research, Yale University, West Haven, CT USA; 3 Center for Rehabilitation Research, VA Connecticut Healthcare System, West Haven, CT USA; 4 Yale Medical Scientist Training Program, Yale School of Medicine, New Haven, CT USA; 5 Interdepartmental Neuroscience Program, Yale School of Medicine, New Haven, CT USA; 6 Department of Clinical Epidemiology and Medical Technology Assessment (KEMTA), Maastricht University Medical Centre, Maastricht, The Netherlands; 7 Department of Clinical Genetics, Maastricht University Medical Centre+, Maastricht, The Netherlands

**Keywords:** Ion channels in the nervous system, Cellular neuroscience, Pain

## Abstract

The inhibition of voltage-gated sodium (Na_V_) channels in somatosensory neurons presents a promising novel modality for the treatment of pain. However, the precise contribution of these channels to neuronal excitability, the cellular correlate of pain, is unknown; previous studies using genetic knockout models or pharmacologic block of Na_V_ channels have identified general roles for distinct sodium channel isoforms, but have never quantified their exact contributions to these processes. To address this deficit, we have utilized dynamic clamp electrophysiology to precisely tune in varying levels of Na_V_1.8 and Na_V_1.9 currents into induced pluripotent stem cell-derived sensory neurons (iPSC-SNs), allowing us to quantify how graded changes in these currents affect different parameters of neuronal excitability and electrogenesis. We quantify and report direct relationships between Na_V_1.8 current density and action potential half-width, overshoot, and repetitive firing. We additionally quantify the effect varying Na_V_1.9 current densities have on neuronal membrane potential and rheobase. Furthermore, we examined the simultaneous interplay between Na_V_1.8 and Na_V_1.9 on neuronal excitability. Finally, we show that minor biophysical changes in the gating of Na_V_1.8 can render human iPSC-SNs hyperexcitable, in a first-of-its-kind investigation of a gain-of-function Na_V_1.8 mutation in a human neuronal background.

## Introduction

Chronic pain affects approximately 100 million American adults^1^ and similarly high proportions of adults globally^2–7^, underscoring the importance of adequate pain management. Unfortunately, current mainstays of pain management are often only partially effective^8,9^, addictive^10^, or are associated with serious adverse effects^11^. There is an urgent need for more effective, specific, and well-tolerated treatments for pain.

Voltage-gated sodium (Na_V_) channels have recently emerged as promising therapeutic targets in the treatment of pain. There are nine Na_V_ channel isoforms (Na_V_1.1-Na_V_1.9)^12,13^, three of which (Na_V_1.7, Na_V_1.8, and Na_V_1.9)^14^ are preferentially expressed in dorsal root ganglion (DRG) neurons, the primary afferents of the peripheral nervous system whose hyperexcitability has been shown to result in pain^15–18^. Additionally, all three of these Na_V_ channels have been genetically and functionally linked to pain disorders in humans^19–26^. Consequently, clinical trials investigating the efficacy of Na_V_ channel blockers in multiple painful disease states have been conducted, although progress has been mixed and no Na_V_1.7, Na_V_1.8, or Na_V_1.9-specific channel blocker has yet been approved for clinical use^27^.

While the functional consequence of Na_V_ isoform mutations is well-understood, knowledge of the precise role of these channels is also helpful in designing channel blockers. Currently, most knowledge concerning the roles of Na_V_1.8 and Na_V_1.9 is based on biophysical studies in heterologous expression systems or derived from knock-out or pharmacologic block studies. For example, Na_V_1.8 is known to contribute to the overshoot and width of DRG neuron action potentials through an all-or-none knockout study^28^. Similarly, Na_V_1.9 displays extensive window current^29^ (i.e., overlap of activation and fast-inactivation) that has been theorized to contribute to the depolarized resting membrane potentials of rodent DRG neurons expressing a gain-of-function Na_V_1.9 variant^30^. However, no study has yet been able to quantify the relationship between varying levels of these currents and their effect on neuronal excitability, the cellular correlate of pain^31^. Moreover, there is a pressing need for these studies to be carried out in human DRG neurons.

Dynamic clamp is an electrophysiological technique that allows for modeled currents, derived from real recordings, to be added to or subtracted in precisely calibrated aliquots from cellular systems with high temporal and voltage fidelity^32^. Previous work has shown that adding Na_V_1.7 conductance to rodent DRG neurons results in a linear reduction in current threshold to action potential firing, whereas subtracting Na_V_1.7 conductance linearly increases the threshold to action potential firing^33^. However, there exist many differences between rodent and human DRG neurons, including in the level of expression of Na_V_1.8- and Na_V_1.9-positive nociceptors^34^. Fortunately, the development of induced pluripotent stem cell (iPSC) technology and the ability to differentiate iPSCs into somatosensory neurons (iPSC-SNs) that recapitulate human pain phenotypes^35,36^ has allowed for the interrogation of Na_V_s in human cell backgrounds.

In this study, we capitalize on methodology that permits differentiation of peripheral somatosensory neurons from human IPSCs, and utilize dynamic clamp electrophysiology to confirm roles for the currents passed by Na_V_1.8 and Na_V_1.9 in these human cells. Using before-and-after recordings in the same human sensory neuron, we quantify the contribution of Na_V_1.8 to the action potential half-width and the sensory neuron’s ability to repetitively fire action potentials. Similarly, we quantify a positive relationship between Na_V_1.9 currents and neuronal resting membrane potential. Moreover, we investigated the interplay between Na_V_1.8 and Na_V_1.9 on neuronal excitability, showing that there exists a range of maximal excitability that tapers off with excessive depolarization. Finally, in a first-of-its-kind analysis, we utilized dynamic clamp electrophysiology to simulate Na_V_1.8 gain-of-function in human sensory neurons, showing that even minor alterations in channel gating result in significant alterations in neuronal excitability.

## Methods

### Differentiation of iPSCs into iPSC-SNs

We have previously identified a subject who underwent sequencing of the *SCN9A* gene, encoding Na_V_1.7, and was found to carry no pathogenic Na_V_1.7 mutations^36^. iPSCs were generated from this phenotypically normal subject (no abnormal pain) as previously described^36^. Differentiation of iPSCs into iPSC-SNs used an eight-week modified Chambers protocol^35,37,38^ (Supplementary Table 1). Medium was changed twice weekly after day 12 through day 56 of differentiation and beyond for electrophysiology experiments; electrophysiological recordings were conducted within 7–14 days after completion of differentiation.

### Immunocytochemistry

Eight-week-old differentiated iPSCs-SNs were stained with sensory neuronal markers. The cells were grown on PDL/laminin- and matrigel-coated glass coverslips and were fixed for 10 min in 4% paraformaldehyde. Following a 30 min incubation in 4% donkey serum, 2% BSA, and 0.1% Triton X-100 in PBS (PBS-T), iPSC-SNs were incubated with primary antibodies in PBS-T overnight at 4 °C (Rabbit polyclonal anti-NeuN, 1:200, ab104225, Abcam; chicken polyclonal anti-Peripherin, 1:200, Aves Labs, Tigard, OR; rabbit polyclonal anti-BRN3A, 1:200, AB5945, Millipore; and mouse monoclonal anti-NaV1.7, 1:250, 75–103, Neuromab). The cells were washed in 0.01 M PBS and incubated with secondary antibodies (Jackson ImmunoResearch Labs) in PBS-T (1:1000) for 2 h at room temperature (Donkey anti-chicken-594, AB_2340375, donkey anti-mouse-647, AB_2340863; and donkey anti-rabbit-488, AB_2313584). Coverslips were mounted on microscope glass slides with Aqua Poly/Mount (Cat#18606, Polysciences). Images were acquired 24 h later using a Nikon Confocal TiE inverted microscope with a Plan Fluor 10 × and an Apo LWD 40x/1.5 WI λS, 0.15–0.19 DIC N2 (Nikon, Melville, NY).

### Digital droplet polymerase chain reaction (ddPCR)

Human iPSC-SNs were processed for RNA extraction using an RNeasy kit (Qiagen, Germantown, MD) according to the manufacturer’s protocol. iPSC-SNs were gently enzymatically detached from the vial they were differentiated in and centrifuged immediately for ddPCR analysis, limiting the time possible for gene expression changes. A total of 135 ng of RNA was reverse transcribed using iScript™ Reverse Transcription Supermix (Bio-Rad, Hercules, CA) according to the manufacturer’s instructions. All reagents and equipment used for ddPCR were from Bio-Rad. Twenty-five μl reactions consisted of 1X ddPCR™ Supermix for probes, 1X target primers/probe mix/FAM, 1X reference primers/probe mix/HEX, which correspond to a final concentration of 900 nM primers and 250 nM probe. All the primers/probe mix were from either Bio-Rad or ThermoFisher Scientific (see supplementary Table 2 for details). The samples were partitioned into 20,000 nanoliter-sized droplets using a Droplet Generator. The emulsion of droplets was transferred into a 96-well plate, heat-sealed with pierceable foil, and amplified in a C1000 Touch Thermal Cycler (Bio-Rad). The cycling protocol starts with a 95 °C enzyme activation step for 10 min, followed by 40 cycles of a two-step cycling protocol (94 °C for 30 s and 60 °C for 1 min). The cycling protocol was followed by an enzyme deactivation step of 98 °C for 10 min. A ramp rate of 2 °C per second was required for each step in the PCR. When cycling was complete, the plate was loaded into the QX200 Droplet Reader (Bio-Rad) and data was analyzed using QuantaSoft™ Software (version 1.6.6, Bio-Rad).

### Whole-cell voltage-clamp recordings from iPSC-SNs

All patch clamp recordings were obtained using an EPC-10 amplifier and the PATCHMASTER program (HEKA Elektronik, Holliston, MA) at room temperature (22–25 °C). Patch pipettes were pulled from borosilicate glass (1.65/1.1 mm, outside diameter/inside diameter, World Precision Instruments, Sarasota, FL) using a Sutter Instruments P-97 puller and had a resistance of 0.7–1.5 MΩ. Extracellular bath solution contained (in mM): 140 NaCl, 20 tetraethylammonium (TEA), 3 KCl, 1 CaCl_2_, 1 MgCl_2_, 10 HEPES, 0.1 CdCl (± 0.001 TTX). The extracellular bath solution was titrated to a pH of 7.3 and the osmolarity was titrated to approximately 320 mOsm with sucrose. Intracellular pipette solution consisted of (in mM): 140 CsF, 10 NaCl, 1.1 EGTA, 10 HEPES, and 20 dextrose. The pH was titrated to 7.3 with CsOH and the osmolarity was also titrated to approximately 320 mOsm.

Total sodium current was recorded in TTX-free extracellular solution. iPSC-SNs were held at − 100 mV and subsequently stepped to potentials between − 80 and + 40 mV in 5 mV increments for 100 ms duration. To measure total TTX-R currents, the same protocol was applied in a nearly identical bath solution, differing only in the presence of 1 μM TTX. To measure Na_V_1.8 currents, iPSC-SNs were held at − 50 mV to inactivate all non-Na_V_1.8 channels^39–43^ and then stepped to potentials, for 100 ms, between − 80 and + 40 mV. Na_V_1.9 currents were considered present if a persistent current with peak current amplitude around − 50 mV was observed after reference series subtraction of the current evoked by holding neurons at − 50 mV from total TTX-R current. No Na_V_1.9 currents were appreciated using this paradigm.

### Isolation and voltage-clamp recordings of primary human autopsy-derived DRG neurons

Human DRG tissues [lumbar 4 (L4) or thoracic 12 (T12)] were received as anonymized samples from the National Disease Research Interchange. Studies with human tissues were approved by the human investigation committee at Yale University and all methods were performed in accordance with the relevant guidelines and regulations. DRGs were recovered from multiple adult (ages 18–70) human donors, of both sexes, with no history of diabetes, neuropathies, cancer, chemotherapy, or radiation. Previous use of antiepileptic, antiarrhythmic, or local anesthetic medications were also exclusion criteria, as well as any history of trauma to the lower limbs or a history of lumbosacral injury or pain. Human DRGs were obtained from organ donors with full legal consent for use of tissue for research. Informed consent was acquired prior to all tissue donation. DRG neurons were harvested and dissociated within 30 h of clamping the aorta.

Human DRG neurons were cultured as previously described^44^. In brief, nerve roots and connective tissue were removed, DRGs were sliced into small pieces in complete saline solution (CSS) [in mM: 137 NaCl, 5.3 KCl, 1 MgCl2, 25 sorbitol, 3 CaCl2, and 10 HEPES, adjusted to pH 7.2 with NaOH], and then incubated on a rotating shaker at 37 °C for 40–60 min in CSS containing 0.5 U/mL Liberase TM (Roche) and 0.6 mM EDTA. This was followed by a 25–40 min (L4 DRG) or 40–60 min (T12 DRG) incubation at 37 °C in CSS containing 0.5 U/mL Liberase TL (Roche), 0.6 mM EDTA, and 30 U/mL papain (Worthington Biochemical). DRGs were then triturated in DRG media [DMEM/F12 (Invitrogen) with 100 U/ml penicillin, 0.1 mg/ml streptomycin (Invitrogen), and 10% fetal bovine serum (Hyclone)] containing 1.5 mg/mL BSA (low endotoxin) and 1.5 mg/mL trypsin inhibitor (Sigma). After filtering with a 100 μm nylon mesh cell strainer (BD Biosciences), the cell suspension was centrifuged and the cell pellet was resuspended in DRG media. 100 μl of cell suspension were plated onto each poly-D-lysine/laminin-coated coverslip (BD Biosciences). Plated DRG neurons were incubated at 37 °C in a 95% air/5% CO_2_ (vol/vol) incubator for 60–90 min to allow neurons to adhere. 900 µL of DRG media supplemented with nerve growth factor (50 ng/mL) and glial cell line-derived neurotrophic factor (50 ng/mL) were added into each well. DRG neurons were maintained at 37 °C in a 95% air-5% CO_2_ (vol/vol) incubator and recorded by whole cell patch clamp between 1- and 4-days post-culture.

Na_V_1.8 currents were recorded from human DRG neurons in a bath solution containing (in mM): 70 NaCl, 70 N-methyl-d-glucamine (NMDG), 20 TEA, 3 KCl, 1 CaCl_2_, 1 MgCl_2_, 10 HEPES, 0.1 CdCl, and 0.001 TTX. The pH was titrated to 7.3 with HCl and the osmolarity was titrated to approximately 320 mOsm with sucrose. Intracellular pipette solution consisted of (in mM): 140 CsF, 10 NaCl, 1.1 EGTA, 10 HEPES, and 20 dextrose. The pH was titrated to 7.3 with CsOH and the osmolarity was also titrated to approximately 320 mOsm. Na_V_1.8 currents were recorded from human DRG neurons by first holding the cells to − 60 mV to inactivate Na_V_1.9 channels. Cells were then stepped to a range of potentials, from − 80 to + 40 mV in 5 mV increments, for 200 ms to record Na_V_1.8 currents. Data were sampled every 20 µs and P/6 leak subtraction was implemented.

### Current-clamp and dynamic-clamp recordings of iPSC-SNs

iPSC-SNs were recorded from within 7–14 days after the completion of the eight-week differentiation protocol, with medium changes twice per week. Extracellular bath solution contained (in mM): 140 NaCl, 3 KCl, 2 CaCl_2_, 2 MgCl_2_, 15 dextrose, and 10 HEPES. Osmolarity was brought to approximately 320 mOsm with sucrose and the pH was titrated to 7.3 with NaOH. Intracellular pipette solution contained (in mM): 140 KCl, 3 Mg-ATP, 0.5 EGTA, 5 HEPES, and 20 dextrose. Osmolarity was similarly adjusted to approximately 320 mOsm and the pH was adjusted to 7.3 using KOH. Current-clamp recordings were sampled at 50 kHz and filtered using two Bessel filters at 10 and 2.9 kHz.

iPSC-SNs with an input resistance lower than 200 MΩ were excluded from analysis. Input resistance was determined by the slope of a linear fit to hyperpolarizing responses to current steps from − 5 pA to − 40 pA in − 5 pA increments. Additionally, neurons with an action potential overshoot below + 40 mV were excluded. Multiple recordings, before and after the addition of currents by dynamic clamp, were taken from each neuron, contingent upon the input resistance remaining above 200 MΩ. Dynamic clamp models of Na_V_1.8 and Na_V_1.9 were derived from published literature; see Han et al.^44^ and Huang et al.^26^ for further information regarding the kinetic models of these channels. iPSC-SNs were dynamically clamped using the Cybercyte DC1 dynamic clamp system (Cytocybernetics, Buffalo, NY)^45,46^.

In brief, the Nav1.8 channel model was based on Hodgkin–Huxley equations $\frac{{{\text{d}}m}}{{{\text{d}}t}} = \alpha_{m} \left( {1 - m} \right) - \beta_{m} m$ and $\frac{{{\text{d}}h}}{{{\text{dt}}}} = \alpha_{h} \left( {1 - h} \right) - \beta_{h} h$, where *m* and *h* represent channel activation and inactivation gates, and α and β are forward and backward rate constants, respectively. These rate constants were voltage-dependent and defined by the following equations:

$$ \alpha_{m} = 7.35 - \frac{7.35}{{1 + e^{{\frac{V + 1.38}{{10.9}}}} }} $$

$$ \beta_{m} = \frac{5.97}{{1 + e^{{\frac{V + 56.43}{{18.26}}}} }} $$

$$ \alpha_{h} = 0.011 + \frac{1.39}{{1 + e^{{\frac{V + 78.04}{{11.32}}}} }} $$

$$ \beta_{h} = 0.56 - \frac{0.56}{{1 + e^{{\frac{V - 21.82}{{20.03}}}} }} $$

Similarly, Na_V_1.9 was modeled with the following equations:

$$ \alpha_{m} = \frac{0.751}{{1 + e^{{\frac{{ - \left( {V + 32.26} \right)}}{13.71}}} }} $$

$$ \beta_{m} = \frac{5.68}{{1 + e^{{\frac{V + 123.71}{{13.94}}}} }} $$

$$ \alpha_{h} = \frac{0.082}{{1 + e^{{\frac{V + 113.69}{{17.4}}}} }} $$

$$ \beta_{h} = \frac{0.24}{{1 + e^{{\frac{{ - \left( {V - 10.1} \right)}}{17.2}}} }} $$

$$ \alpha_{s} = \frac{0.019}{{1 + e^{{\frac{V + 154.51}{{11.46}}}} }} $$

$$ \beta_{s} = \frac{0.000376}{{1 + e^{{\frac{{ - \left( {V + 60.92} \right)}}{15.79}}} }} $$

In the case of the Nav1.9 channel, a slow-inactivation gate (*s* variable) was included and modeled using the $$\frac{{{\text{ds}}}}{{{\text{d}}t}} = \alpha_{s} \left( {1 - s} \right) - \beta_{s} s$$. Nav1.8 current was described by $$I_{Na} = g_{max} *m^{3} *h*\left( {V_{m} - E_{Na} } \right)$$. Nav1.9 current was described by the equation $$I_{Na} = g_{max} *m*h*s*\left( {V_{m} - E_{Na} } \right)$$, where *g*_*max*_ is the maximal conductance and E_Na_ is the reversal potential, which was set to + 65 mV.

Current threshold was defined as the first current injection step that resulted in action potential firing without subsequent failure and was determined by a series of depolarizing current injections (200 ms) that increased incrementally by 5 pA. For the calculation of threshold, action potentials were defined as rapid increases in membrane potential to > 40 mV with a total amplitude > 80 mV. However, as neurons often attenuate firing with repetitive action potential spiking, when examining repetitive firing, action potentials were counted if the membrane potential rapidly crossed 0 mV, regardless of overshoot or total amplitude. Action potential repetitive firing was determined by summing the total number of action potentials that a neuron fired after a 500 ms current injection.

### Data analysis and visualization

Data were analyzed and visualized in GraphPad Prism and Matlab (for three-dimensional surface fitting). Significance in figures is noted as * (*p* ≤ 0.05), ** (*p* ≤ 0.01), or *** (*p* ≤ 0.001). Paired Student’s t-tests were conducted when comparing before-and-after recordings from the same neurons. Best-fit equations are graphed with a solid line. Individual points on graphs represent individual recordings, although multiple recordings were conducted in cells that continued to meet inclusion criteria (input resistance at least 200 MΩ and an action potential amplitude, measured from resting potential to peak, of > 80 mV). Hashed lines on graphs represent the 95% confidence interval for fitted equations.

## Results

### Generation and characterization of iPSC-SNs

**Figure 1:**
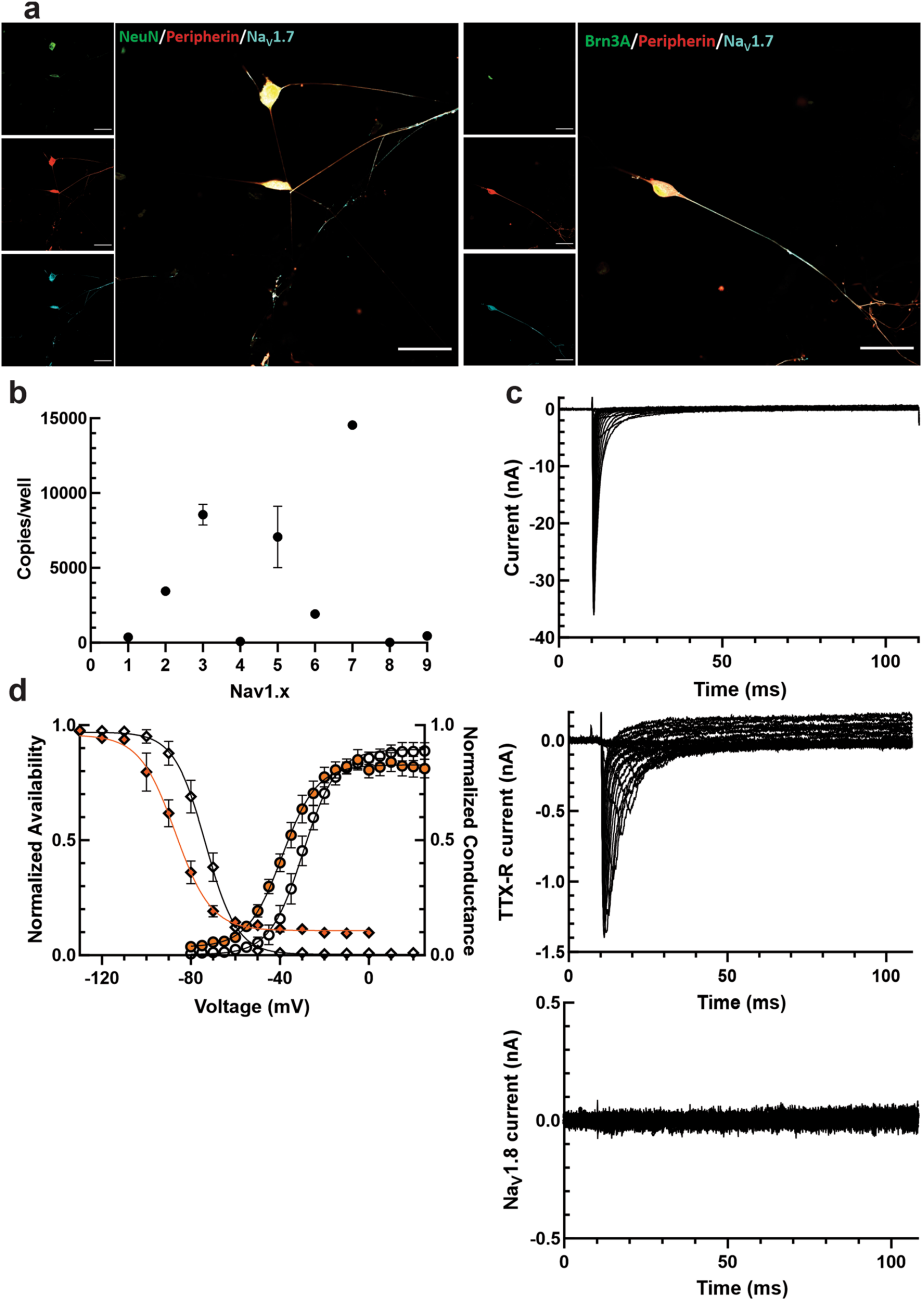
Generation and characterization of iPSC-SNs. (**A**) iPSC-SNs express canonical sensory neuronal markers. 50 µm scale bar for reference. (**B**) iPSC-SNs express very high levels of Na_V_1.7 mRNA, but virtually no levels of Na_V_1.8 and Na_V_1.9 mRNA, as determined by droplet digital PCR. (**C**) Representative current traces recorded from iPSC-SNs confirm large total sodium currents (top). However, application of 1 µM tetrodotoxin reveals very little tetrodotoxin-resistant current, which behaves like Na_V_1.5 and not Na_V_1.8 or Na_V_1.9 (middle). Consistent with the lack of Na_V_1.8 expression by PCR analysis, there is also no Na_V_1.8 current when cells are clamped at a holding potential of − 50 mV to inactivate all other sodium channels besides Na_V_1.8 (bottom). (**D**) The V_1/2_ of activation (open circles) and fast-inactivation (open diamonds) of total sodium current in iPSC-SNs was − 29.83 ± 2.43 mV and − 74.08 ± 2.55 mV, respectively. The V_1/2_ of activation (orange circles) and fast-inactivation (orange diamonds) of TTX-R sodium currents in iPSC-SNs was − 38.17 ± 1.80 mV and − 87.79 ± 2.93 mV, respectively.

All iPSCs were differentiated from a previously identified subject with no abnormal pain and no Na_V_1.7 channel mutations^36^ into iPSC-SNs using a modified Chambers protocol that produces pain-sensing sensory-like neurons^35,37,47^ (Supplementary Table 1). The differentiated cells were validated as peripheral somatosensory neurons by immunocytochemistry (Fig. 1A). iPSC-SNs stained positively for peripherin (a marker of peripheral neurons), Brn3a (a marker of sensory neurons^48^), NeuN (a marker of neuronal nuclei), and Na_V_1.7. Unpublished work from our lab and others in this field has questioned whether current iPSC-SN differentiation protocols are able to express the TTX-R Na_V_ channels, Na_V_1.8 and Na_V_1.9, seen in human DRG neurons. In line with these previous observations, we were unable to show any Na_V_1.8 or Na_V_1.9 RNA by ddPCR (Fig. 1B) or current by whole-cell voltage-clamp electrophysiology (Fig. 1C); conversely, the iPSC-SNs expressed very high levels of Na_V_1.7, as would be expected from somatosensory neurons. TTX-R currents observed in iPSC-SNs exhibited a very hyperpolarized V_1/2_ of activation (− 38.17 ± 1.80 mV, n = 8) and fast-inactivation (− 87.79 ± 2.93 mV, n = 5, Fig. 1D), consistent with previous characterizations of iPSC-SNs and closer to values for Na_V_1.5 than for Na_V_1.8^49^, suggesting that these cells displayed some characteristics of immature somatosensory-like neurons. Evaluation of iPSC-SNs baseline characteristics by current-clamp displayed largely normal properties with an average cell capacitance of 27.09 ± 1.25 pF, an average input resistance of 320.07 ± 16.2 MΩ, and an average resting membrane potential of − 57.25 ± 0.41 mV (n = 71).

The interesting absence of Na_V_1.8 and Na_V_1.9 from the differentiated iPSC-SNs allows for introduction of precisely calibrated levels of these currents by dynamic clamp. Rather than first voltage-clamping neurons to measure the current levels (which may run up or down during experimentation^50–53^), and then subtracting out the measured—and adding back in the modeled—TTX-R currents, we are able to know precisely how much Na_V_1.8 and Na_V_1.9 current the neurons are conducting since the amplitude of current is precisely controlled by dynamic clamp.

### Na_V_1.8 contributes to action potential overshoot, half-width, and repetitive firing

**Figure 2:**
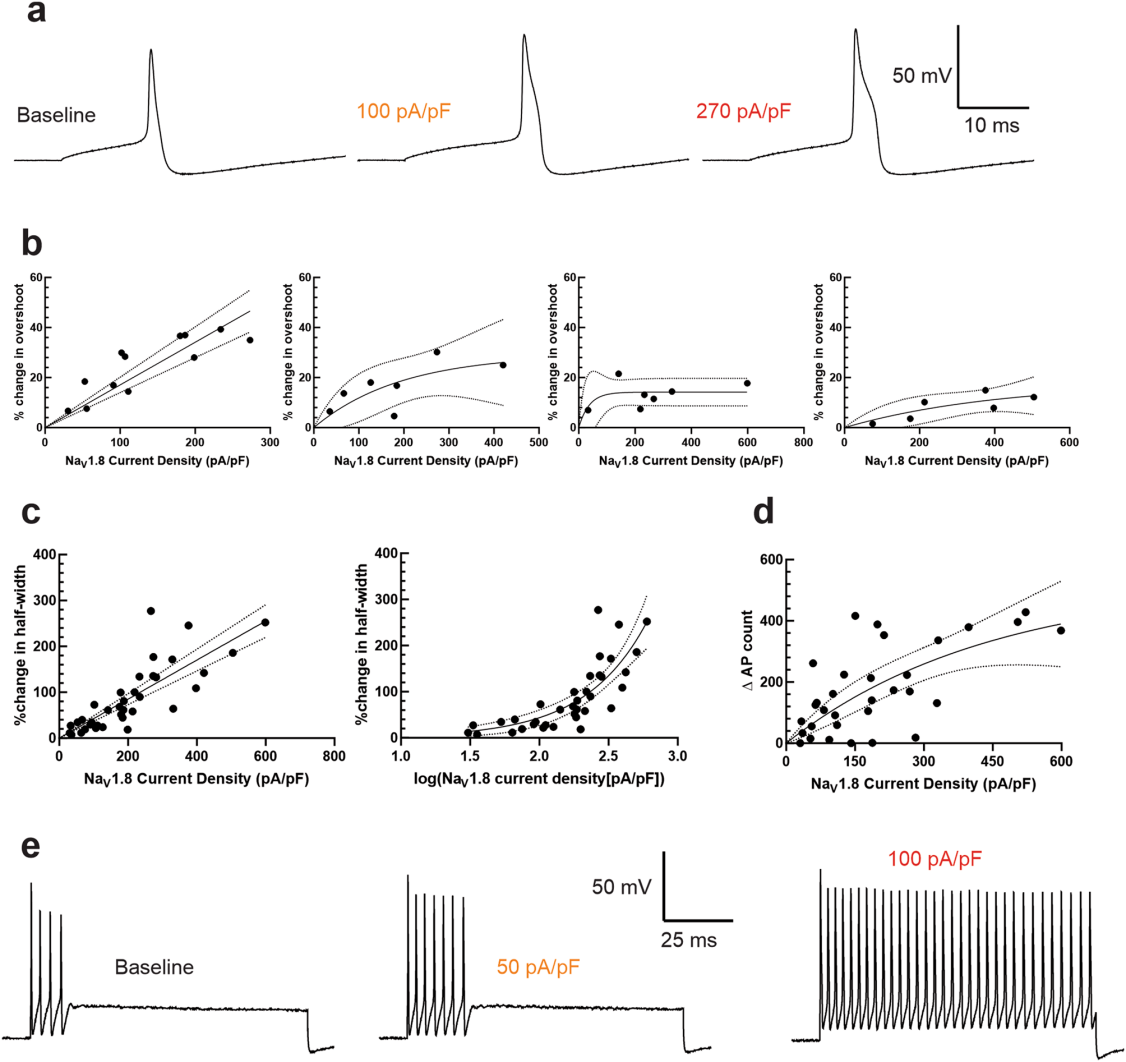
Na_V_1.8 contributes to action potential overshoot, half-width, and repetitive firing. (**A**) Representative traces from the same iPSC-SN illustrating the action potential waveform in the setting of varying levels of Na_V_1.8 current density. (**B**) Increasing Na_V_1.8 current density increases the overshoot of iPSC-SNs, although the effect is more robust at lower initial overshoot amplitudes. For neurons with an initial overshoot amplitude between 40 and 45 mV (far left), the change in overshoot is best fit with a linear model with slope 0.1706 and an r^2^ of 0.5093. For neurons with an initial overshoot between 45 and 50 mV (center-left), 50–55 mV (center-right), and 55–60 mV (far right), the change in overshoot amplitudes are best fit with exponential association equations. (**C**) Increasing Na_V_1.8 current density directly increases the action potential half-width of iPSC-SNs linearly (% change in half-width = 0.4254*current density) with an r^2^ of 0.65. An equivalent transformation of the data into base-10 logarithmic form illustrates a similarly robust relationship (% change in half-width = 0.4816*e^2.253(log[current density])^) with an r^2^ of 0.6502. (**D**) Increasing Na_V_1.8 current density enhances iPSC-SN repetitive firing following (Δ action potential count = 524.9*(1 − e^−0.002266(current density)^) with an r^2^ of 0.4164. (**E**) Representative traces depicting the response of the same iPSC-SN to a 1 s duration 500 pA suprathreshold stimulus with no Na_V_1.8 currents injected via dynamic clamp (left), approximately 50 pA/pF Na_V_1.8 current density (middle), and 100 pA/pF current density (right).

To investigate the effects of Na_V_1.8 currents on parameters of cellular excitability, we tuned in varying levels of Na_V_1.8 current density using a previously published kinetic model of the human Na_V_1.8 channel^44^ and the CytoCybernetics Cybercyte dynamic clamp system. The peak Na_V_1.8 current density injected was determined by normalizing the peak modeled current (determined by a series of 100 ms pulses from a holding potential of − 100 mV to + 40 mV) to the cell capacitance of the patched iPSC-SN. Representative traces of iPSC-SNs with various levels of modeled Na_V_1.8 current visualize an effect of increasing this current density on neuronal action potentials (Fig. 2A).

As we controlled the precise amount of Na_V_1.8-like current passing through the cell, we next sought to quantify the effect of this conductance on neuronal excitability and electrogenesis properties. First, as Renganathan et al. showed, Na_V_1.8 plays an important role in contributing to the overshoot of the action potential^28^, likely due to this channel’s depolarized voltage-dependences of activation and inactivation^54^. We observed this visually, but quantifying this parameter proved to be difficult as the action potential overshoot cannot exceed the Nernst potential of approximately + 67 mV^55^. Indeed, the absolute maximum overshoot observed in our study was + 68 mV. We attempted to best quantify the change in action potential overshoot by stimulating neurons with 200 ms long square pulses of increasing current amplitude (from 5 pA) in 5 pA increments, until the current threshold was reached. We then measured the action potential overshoot at threshold before and after injection of Na_V_1.8 current by dynamic clamp (Fig. 2B). As the ability for the action potential overshoot to increase is diminished as the initial overshoot nears the Nernst potential for sodium, we have binned the data in 5 mV increments, based on the overshoot amplitude prior to dynamic clamp injection. There is a clear linear relationship (slope = 0.1706, r^2^ = 0.59) between Na_V_1.8 current density and action potential overshoot in neurons whose baseline overshoot was between + 40 and + 44.99 mV (Fig. 2B, left), which represented a plurality of the recordings (n = 12). This relationship becomes less obvious as the baseline overshoot is increased.

To quantify the observed effect of Na_V_1.8 currents on action potential half-width, a similar paradigm was implemented as above. The half-width was determined as the duration of time between the rising phase and falling phase of the action potential at the point midway between the overshoot and the undershoot. We observed a linear relationship between Na_V_1.8 current density and action potential half-width (Fig. 2C, left), with a slope of 0.4254 and an r^2^ of 0.65. This relationship was similarly strong when current density was transformed into the base-10 logarithmic form (Fig. 2C, right).

Additionally, Na_V_1.8 exhibits rapid recovery from inactivation^56^, which has been thought to contribute to repetitive action potential firing in DRG neurons. To quantify this contribution, we stimulated DRG neurons with square pulses of 500 ms duration that increased in amplitude between 25 and 500 pA in 25 pA increments. We summed up the total number of action potentials fired under this protocol before and after injection of Na_V_1.8 modeled currents by dynamic clamp (Fig. 2D). We noted a direct relationship between Na_V_1.8 current density and action potential repetitive firing, with r^2^ of 0.4164. Representative traces (Fig. 2E) illustrate the effect of varying the Na_V_1.8 peak current density injected via dynamic clamp on the same iPSC-SN when stimulated by a 500 pA suprathreshold stimulus. We also recorded Na_V_1.8 currents from human DRG neurons (Fig. 5B,C). If iPSC-SNs lacking endogenous Na_V_1.8 currents expressed a similar level as autopsy-derived human DRG neurons (~ 290 pA/pF), we would expect that these iPSC-SNs would fire approximately 250 more action potentials across our range of stimuli and would have a half-width approximately 120% as wide.

### Increasing Na_V_1.9 currents depolarize the resting membrane and reduce the threshold to action potential firing

**Figure 3:**
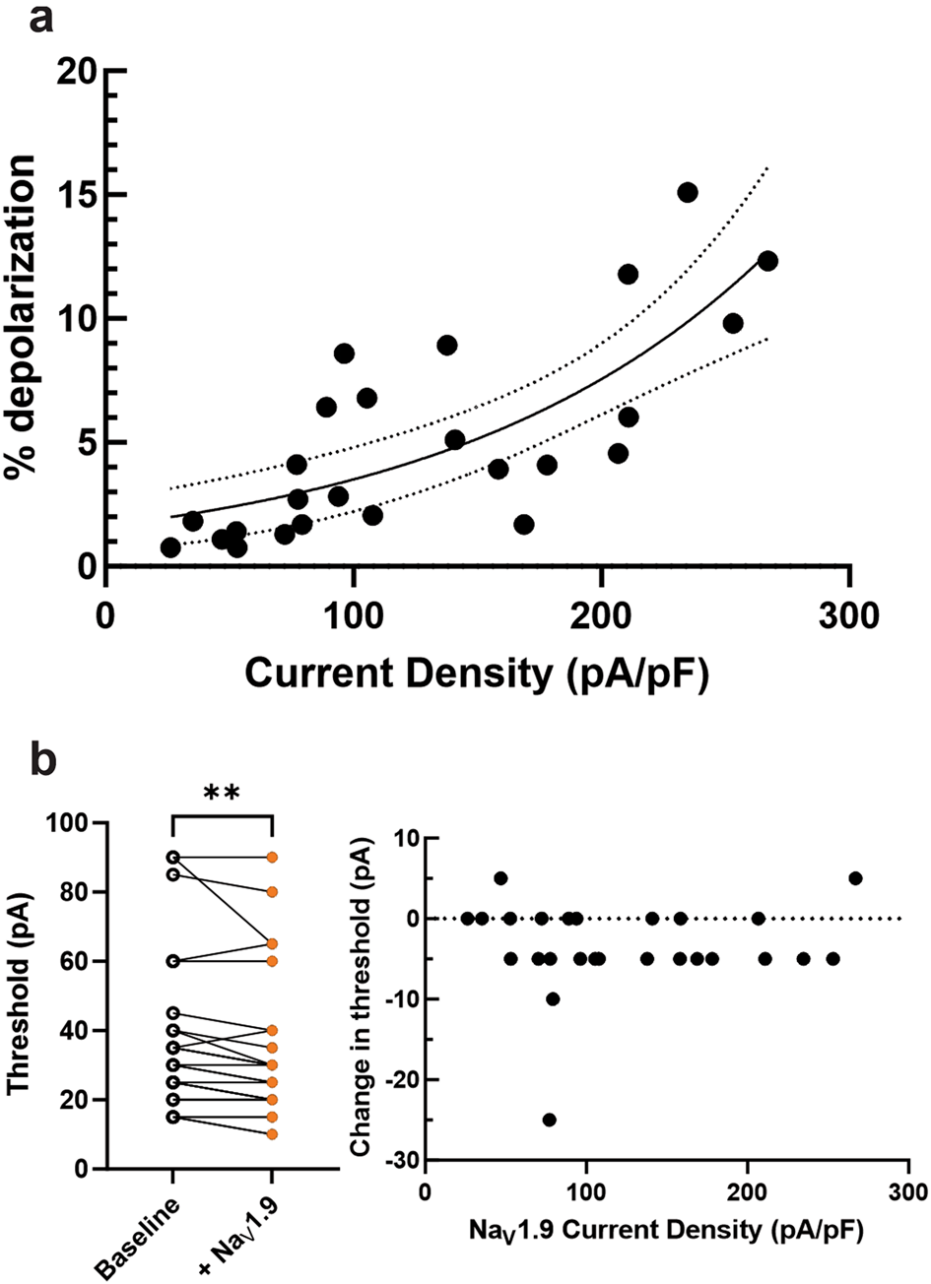
Na_V_1.9 is responsible for setting the neuronal resting membrane potential and plays a role in setting the threshold to action potential firing. (**A**) Increasing Na_V_1.9 current density depolarizes iPSC-SN resting membrane potentials (% depolarization = 1.629*e^0.007674(current density)^) with an r^2^ of 0.5764. (**B**) Adding Na_V_1.9 currents to iPSC-SNs results in a statistically significant reduction in threshold to action potential firing (left, paired t-test *p* = 0.0043). However, there does not appear to be a strong correlation between the levels of Na_V_1.9 current density and the change in threshold (right).

We then investigated the role of Na_V_1.9 in neuronal excitability. Our primary focus was on the Na_V_1.9’s ability to set and depolarize the neuronal membrane potential. Because of extensive overlap between the activation and inactivation curves of the channel, Na_V_1.9 passes significant amounts of “window current,” and gain-of-function mutations have been linked to depolarized resting potentials in DRG neurons^26^. To quantify the ability of Na_V_1.9 currents to set the resting membrane potential, we averaged 30 s of passive membrane potential with no stimulus before addition of any modeled Na_V_1.9 current density and compared that average membrane potential, in the same neuron, after addition of modeled Na_V_1.9 currents by dynamic clamp (Fig. 3A). We observed a positive correlation between Na_V_1.9 current density and membrane potential depolarization (which ranged from 0 mV with no addition of Nav1.9 current, to 9.7 mV with addition of 235 pA/pF Nav1.9 current), and, when fit with an exponential growth equation, the r^2^ of the fit was 0.5764.

The addition of Na_V_1.9 current density to iPSC-SNs depolarized their resting membrane potential, theoretically bringing them closer to the voltage threshold for action potential firing. Therefore, we sought to quantify whether there was a relationship between Na_V_1.9 current density and the current stimulus required for action potential firing. iPSC-SNs with Na_V_1.9 currents by dynamic clamp displayed a significantly reduced threshold to action potential firing (Fig. 3B left, paired Student’s t-test *p* = 0.0043, n = 26). However, there appeared to be no relationship between the amount of Na_V_1.9 current density and the reduction in current threshold (Fig. 3B, right).

### Na_V_1.8 and Na_V_1.9 work together to contribute to repetitive firing of DRG neurons

**Figure 4:**
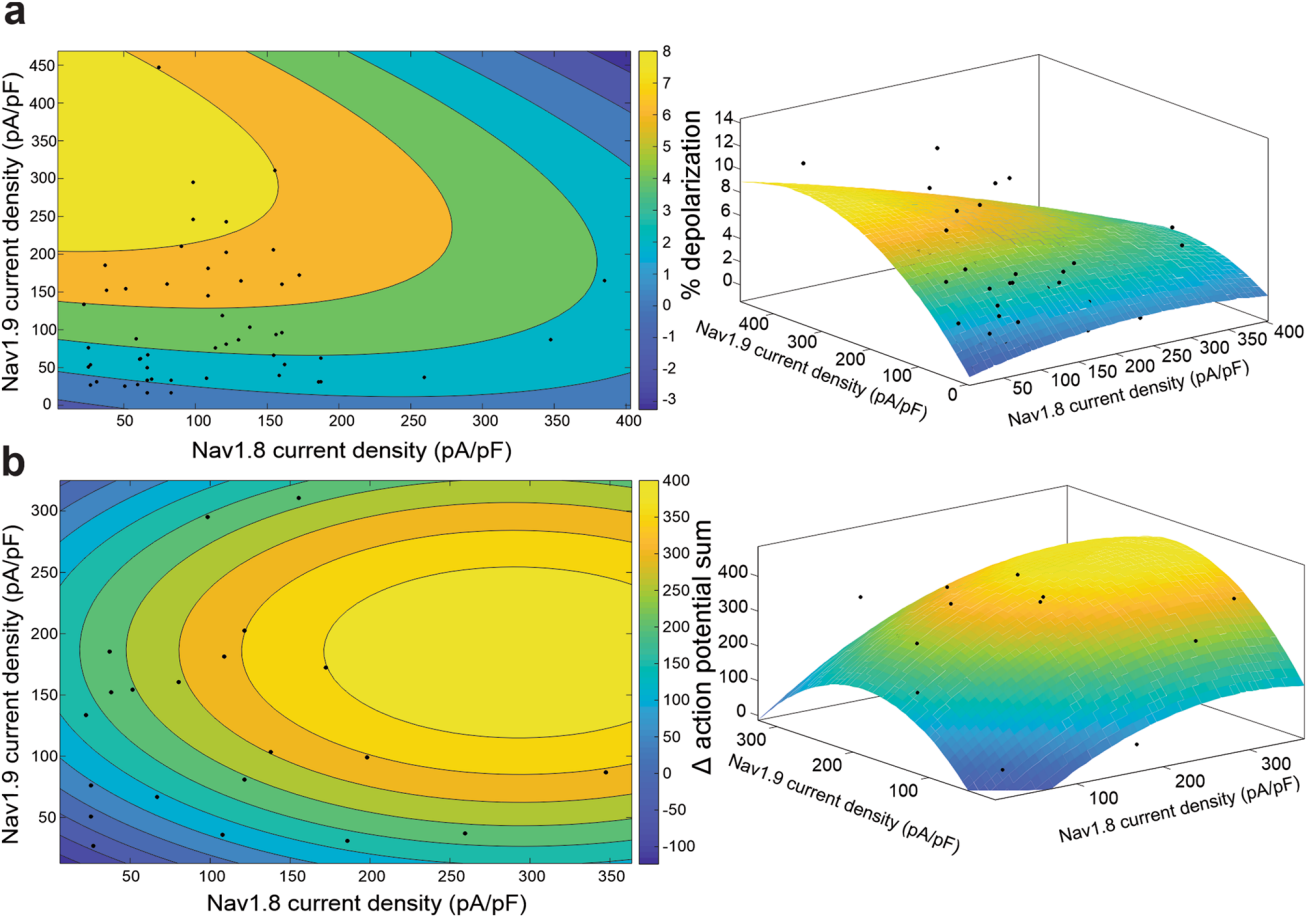
While the resting membrane potential is primarily set by Na_V_1.9, both Na_V_1.9 and Na_V_1.8 play important roles in repetitive firing. (**A**) The relationship between Na_V_1.8 and Na_V_1.9 current densities with the change in iPSC-SN resting membrane potential can be approximated with a 3-dimensional polynomial curve with two degrees of freedom for the x- and y-axes (right, adjusted r^2^ = 0.4147). A contour plot (left) of this fit illustrates that Na_V_1.9 current density has a stronger effect on changing the neuronal resting membrane potential. (**B**) The relationship between Na_V_1.8 and Na_V_1.9 current densities with the change in iPSC-SN repetitive firing can be approximated with a 3-dimensional polynomial curve with two degrees of freedom for the x- and y-axes (right, adjusted r^2^ = 0.3514). A contour plot of this fit illustrates that both Na_V_1.8 and Na_V_1.9 contribute to enhanced repetitive firing.

Because human DRG neurons may express both Na_V_1.8 and Na_V_1.9, we then investigated the interplay between currents from these two channels on parameters of neuronal excitability. First, we varied Na_V_1.8 and Na_V_1.9 current densities and evaluated the depolarization of the resting membrane potential in response to these alterations (Fig. 4A). When fit with a polynomial with two degrees of freedom for the x variable and two degrees of freedom for the y variable (to avoid overfitting), our model indicated that Na_V_1.9 currents are predominantly responsible for changing the neuronal membrane potential. The adjusted r^2^ of the fit was 0.4147 and coefficient values for the fit are located in Table 1. At potentials more negative than − 50 mV, the window current created by the overlap of activation and inactivation allows for the depolarizing effect of the Na_V_1.9 currents on neuronal resting membrane potential, although the effect observed here may be limited by inactivation as the membrane potential depolarizes further^29^. However, no iPSC-SNs patched in this study had a resting membrane potential more positive than − 49.67 mV and the average membrane potential was approximately − 57 mV.

**Table 1:** Parameters of the polynomial fit of Na_V_1.8 and Na_V_1.9 current density on neuronal membrane potential.

Coefficients	Value	95% confidence bounds
f(x,y) = p00 + p10*x + p01*y + p20*x^2^ + p11*x*y + p02*y^2^		
p00	− 0.4887	(− 3.214, 2.237)
p10	0.0159	(− 0.01317, 0.04496)
p01	0.05811	(0.02557, 0.09064)
p20	− 3.091 × 10^−5^	(− 0.0001129, 5.11 × 10^−5^)
p11	− 7.22 × 10^−5^	(− 0.0002453, 0.0001009)
p02	− 8.082 × 10^−5^	(− 0.0001426, − 1.908 × 10^−5^)

We also investigated the effect of dual Na_V_1.8 and Na_V_1.9 currents on repetitive firing in human iPSC-SNs (Fig. 4B). When also fit with a polynomial with two degrees of freedom for the x and y variables, the adjusted r^2^ of the fit was 0.3514. Interestingly, the model predicts a peak “zone” of excitability in the interplay between Na_V_1.8 and Na_V_1.9; as Na_V_1.9 current density increases, the ability of a neuron to repetitively fire increases, up to a point, until excitability again begins to dampen—creating an “inverted u-shaped”^57^ model for neuronal excitability, which likely reflects reduced availability of Na_V_1.7 as increased levels of Na_V_1.9 depolarize the neuronal membrane potential. The coefficient values for the fit can be found in Table 2.

**Table 2:** Parameters of the polynomial fit of Na_V_1.8 and Na_V_1.9 current density on repetitive action potential firing.

Coefficients	Value	95% confidence bounds
f(x,y) = p00 + p10*x + p01*y + p20*x^2^ + p11*x*y + p02*y^2^		
p00	− 180.8	(− 448.4, 86.75)
p10	1.967	(− 0.8241, 4.758)
p01	3.708	(0.8919, 6.524)
p20	− 0.003327	(− 0.01024, 0.003586)
p11	− 0.0001459	(− 0.01556, 0.01527)
p02	− 0.009923	(− 0.01862, − 0.00123)

### Small biophysical changes in Na_V_1.8 significantly alter excitability of human neurons

**Figure 5:**
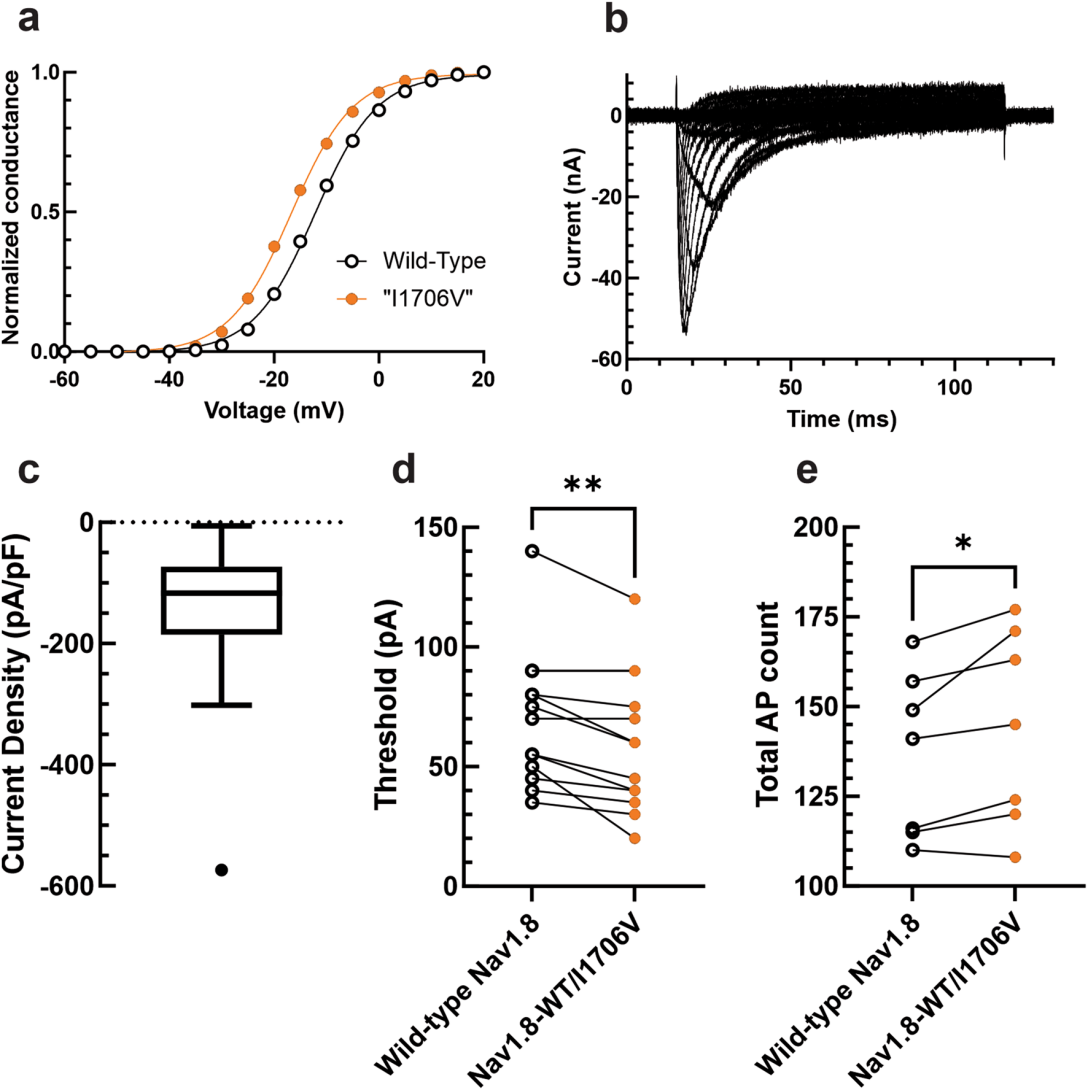
Small biophysical shifts in Na_V_1.8 gating can substantially alter neuronal excitability. (**A**) Conductance-voltage relationship between the wild-type Na_V_1.8 dynamic clamp model and the model with a 4.5 mV hyperpolarized voltage-dependence of activation, approximating the gain-of-function mutation Na_V_1.8-I1706V. (**B**) Representative traces of Na_V_1.8 current recorded from human autopsy-derived DRG neurons. (**C**) Box-and-whisker plot showing the Na_V_1.8 current density recorded from autopsy-derived human DRG neurons in 70 mM NaCl (− 143.69 ± 24.54 pA/pF, n = 24). (**D**) Injection of 50% wild-type Na_V_1.8 current density and 50% “Na_V_1.8-I1706V” current density resulted in a statistically significant (paired t-test *p* = 0.0023, n = 13) reduction in current threshold to action potential firing. (**E**) Injection of 50% wild-type Na_V_1.8 current density and 50% “Na_V_1.8-I1706V” current density resulted in a statistically significant (paired t-test *p* = 0.0367, n = 7) enhancement of repetitive action potential firing.

Having shown that variations in TTX-R current levels result in significant changes in neuronal excitability, we next investigated whether variations in gating properties also result in appreciable alterations in excitability. The Na_V_1.8-I1706V small-fiber neuropathy-associated variant displays a hyperpolarized V_1/2_ of activation, approximately 6 mV more hyperpolarized than wild-type, with no change in the voltage-dependence of fast-inactivation^58^. To roughly approximate this mutation, we equally hyperpolarized the activation of our Na_V_1.8 dynamic clamp model (Fig. 5A). Rather than tune in arbitrary amounts of Na_V_1.8 current density, we determined the approximate Na_V_1.8 current levels expressed in human neurons by voltage-clamping autopsy-derived human DRG neurons. To isolate TTX-R channels from the full complement of ion channels expressed in human DRG neurons, extracellular bath solution contained 1 μM TTX, 20 mM TEA, 0.1 mM CdCl_2_, and intracellular pipette solution contained 140 mM CsF. Additionally, Na_V_1.8 was isolated from Na_V_1.9 by holding DRG neurons at − 60 mV, which inactivates the Na_V_1.9 channels. To improve voltage control, extracellular sodium was reduced to 70 mM (see representative traces in Fig. 5B). Human Na_V_1.8 current density was recorded (Fig. 5C). The average recorded value was doubled, to correct for the reduced sodium bath solution, and tuned into iPSC-SNs via dynamic clamp. Outliers were not excluded given that human DRG neurons represent a heterogeneous population of cells.

Na_V_1.8 current was first tuned into iPSC-SNs as 100% wild-type Na_V_1.8. In the same neuron, 50% of the Na_V_1.8 current density was replaced with 50% “Na_V_1.8-I1706V” current density. First, we assessed the effect of the Na_V_1.8 gain-of-function change on threshold to action potential firing (Fig. 5D). When before-and-after recordings were compared, there was a statistically significant reduction in the action potential threshold (paired t-test *p* = 0.0023, n = 13). We then evaluated the effect of this hyperpolarization of activation on the ability of iPSC-SNs to repetitively fire action potentials (Fig. 5E). Following a similar paradigm as above, in which we summed the total action potentials fired by iPSC-SNs after subsequent graded 500 ms depolarizations of increasing stimulus in 25 pA increments, there was a statistically significant increase in action potentials fired with the replacement of 50% Na_V_1.8 current with the hyperpolarization-shifted variant (paired t-test *p* = 0.0367, n = 7).

## Discussion

Taken together, the studies performed here display the utility of dynamic clamp in iPSC-SNs to probe somatosensory neuronal physiology and investigate the pathophysiology of human disease. We have quantified the contribution of the TTX-R Na_V_s, Nav1.8 and Nav1.9, to neuronal physiology in a way that is more precise than pharmacologic approaches and more feasible than attempts at genetic regulation. Furthermore, we have shown that dynamically clamping iPSC-SNs allows for investigation into human pain-related mutations and gain-of-function changes in ion channels.

Most previous studies investigating mutations in various Na_V_ channels have relied on channel transfection and uncontrolled overexpression in a nonhuman cell background—either containing native rodent channels or in a channel-null background. More recent studies utilizing human iPSC-SNs have been unable to probe mutations in Na_V_1.8 due to the inability to differentiate neurons expressing this channel^49^. Dynamically clamping iPSC-SNs has three advantages that further our ability to research human physiology and disease-relevant pathophysiology. First, dynamic clamp circumvents the problem of inability to express the channel genetically by allowing researchers to create kinetic models that mimic its function with high fidelity. Second, dynamic clamp allows for before-and-after recordings in the same neuron, limiting inter-population heterogeneity between samples used in comparisons. Third, dynamically clamping neurons with precise current inputs can be more physiologically relevant than transfection and overexpression. In this study, we calibrated the amount of Na_V_1.8 currents we injected when investigating a Na_V_1.8 gain-of-function mutation to the amount of Na_V_1.8 currents recorded from human autopsy-derived DRG neurons.

While dynamically clamping iPSC-SNs opens up a new avenue for the further study of human physiology and preclinical pharmacology, the technique is not without limitations. First, kinetic models of ion channels for use in dynamic clamp are only as good as the data used to generate said models. It is imperative that the voltage-clamp recordings used to create the models reflect channel biology as relevantly as possible. For example, the equations defining sodium channel gating voltage-dependences and kinetics should ideally contain information reflecting not only activation and fast-inactivation, but also slow-inactivation. When DRG neurons are held at their resting membrane potential (approximately − 50 to − 70 mV), an appreciable amount of slow-inactivation builds up, resulting in reduced channel availability, potentially altering neuronal excitability^59,60^. In the studies conducted here, the Na_V_1.9 kinetic model accounted for slow-inactivation, whereas the Na_V_1.8 model did not, potentially limiting our conclusions. Additionally, another potential limitation of dynamic clamp is that it recapitulates the currents of the ion channels, but not the physical presence of the channel on the membrane. This precludes the binding of channel partners, which are known to play important roles in modulating sodium channels^61,62^ and other ion channels^63^, and therefore does not reflect a completely physiological situation.

Having now shown the utility of dynamically clamping iPSC-SNs, future studies should extend this technique to studying pharmacologic blockade of Na_V_s. Previous studies have shown that iPSC-SNs derived from patients suffering from inherited erythromelalgia respond strongly to Na_V_1.7-specific channel blockers, including PF-05089771^35^. However, the results of clinical trials of this Na_V_1.7 blocker and others have been mixed^64,65^. One potential reason for this mismatch between preclinical and clinical data is the that the level of blockade possible in vivo is less than the pharmacological blockade possible in vitro due to limitations in bioavailability and penetration into the central nervous system^27^. Dynamic clamp of iPSC-SNs could enable understanding of the precise degree of channel inhibition necessary to induce relief from pain without resulting in total loss of helpful and informative pain sensation.

## Supplementary Information

Supplementary Information.

## Data Availability

All data supporting the findings of this study are available from the corresponding author upon reasonable request.
