# Supplementary material for: Contributions of NaV1.8 and NaV1.9 to excitability in human induced pluripotent stem-cell derived somatosensory neurons
Source: Sci Rep. 2021 Dec 20;11:24283. doi: 10.1038/s41598-021-03608-x (PMC8688473; doi:10.1038/s41598-021-03608-x)
Supplement: Supplementary file 1 — Supplementary Information. [file 41598_2021_3608_MOESM1_ESM.docx]

**Contributions of Na_V_1.8 and Na_V_1.9 to excitability in human induced pluripotent stem-cell derived somatosensory neurons**

Matthew Alsaloum^1,2,3,4,5^, Julie I.R. Labau^1,2,3,6,7^, Shujun Liu^1,2,3^, Mark Estacion^1,2,3^, Peng Zhao^1,2,3^, Fadia Dib-Hajj^1,2,3^, Stephen G. Waxman^1,2,3*^

^1^Department of Neurology, Yale University School of Medicine, New Haven, CT, USA.

^2^Center for Neuroscience & Regeneration Research, Yale University, West Haven, CT, USA.

^3^Center for Rehabilitation Research, VA Connecticut Healthcare System, West Haven, CT, USA.

^4^Yale Medical Scientist Training Program, Yale School of Medicine, New Haven, CT, USA.

^5^Interdepartmental Neuroscience Program, Yale School of Medicine, New Haven, CT, USA.

^6^Department of Clinical Epidemiology and Medical Technology Assessment (KEMTA), Maastricht University Medical Centre, Maastricht, The Netherlands.

^7^Department of Clinical Genetics, Maastricht University Medical Centre+, Maastricht, The Netherlands.

* Corresponding author; email: Stephen.waxman@yale.edu

*Supplementary Table 1*. iPSC-SN differentiation protocol.

| Day | Media components + inhibitors | Notes |
| --- | --- | --- |
| -1 | mTeSR + Ri | Ri = 10 µM Y-27632 ROCK inhibitor |
| 0-1 | KSR + LSB | Passage on day 0;  LSB = 100 nM LDN-193189, 10 µM SB-431542  KSR = KnockOut Serum Replacement |
| 2-3 | KSR + LSB + 3i | 3i = 3 µM CHIR99021, 10 µM SU5402, 10 µM DAPT |
| 4-5 | KSR:N2 (3:1) + LSB + 3i | KSR + N2 supplement in a 3:1 ratio |
| 6-7 | KSR:N2 (1:1) + 3i | KSR + N2 supplement in a 1:1 ratio |
| 8-9 | KSR:N2 (1:3) + 3i | KSR + N2 supplement in a 1:3 ratio |
| 10-11 | N2 + 3i | After day 12, change medium 2X/week |
| 12-56 | N2 + 4GF | 4GF = 25 ng/mL BDGN, GDNF, NGF, and NT-3  Passage on day 56 |

*Supplementary Table 2*. Assay probes for ddPCR of human iPSC-SNs

| Gene | Company | Catalog number | Assay ID |
| --- | --- | --- | --- |
| *SCN1A* | Bio-Rad | 100-31225 | qHsaCIP0027254 |
| *SCN2A* | Thermo Fisher | 4331182 | Hs01109871_m1 |
| *SCN3A* | Thermo Fisher | 4331182 | Hs00366913_m1 |
| *SCN4A* | Bio-Rad | 100-31225 | qHsaCIP0030523 |
| *SCN5A* | Thermo Fisher | 4331182 | Hs00165693_m1 |
| *SCN8A* | Thermo Fisher | 4331182 | Hs00274075_m1 |
| *SCN9A* | Bio-Rad | 100-31225 | qHsaCIP0027256 |
| *SCN10A* | Thermo Fisher | 4351372 | Hs01045146_m1 |
| *SCN11A* | Bio-Rad | 100-31225 | qHsaCIP0030516 |
| *HPRT1* | Bio-Rad | 100-31225 | qHsaCIP0030549 |
